# Comparison of postoperative hypersensitivity between Total-etch and Universal adhesive system: a randomized clinical trial

**DOI:** 10.1038/s41598-024-51175-8

**Published:** 2024-01-05

**Authors:** Kiran Javed, Nouman Noor, Muhammad Zubair Nasir, Manzoor Ahmed Manzoor

**Affiliations:** 1 Department of Operative Dentistry & Endodontics, Rawal Institute of Health Sciences, Lehtrar Road, Islamabad, 44000 Pakistan; 2 Department of Operative Dentistry & Endodontics, School of Dentistry, Shaheed Zulfiqar Ali Bhutto Medical University, Islamabad, 44000 Pakistan; 3 Department of Community Dentistry, Shifa College of Dentistry, Shifa Tameer-e-Millat University, Islamabad, 44000 Pakistan

**Keywords:** Dentistry, Prognosis, Therapeutics

## Abstract

The objective was to determine the postoperative hypersensitivity of two-step Total-etch as compared to one-step Universal adhesives followed by composite restorations on 100 patients by applying Total-etch on one tooth and Universal adhesive on another tooth. The bonds and teeth were randomly selected. Postoperative hypersensitivity was recorded by visual analog scale before, immediately after, and 24 h after the restoration using cold stimulus. The Mann–Whitney test was applied for statistical comparison of postoperative hypersensitivity between the two bonds as well as for any significant difference in genders with each bond. No significant difference was found between postoperative hypersensitivity of the two adhesives before (p-value = 0.57), immediately after (p-value = 0.604), and 24 h after (p-value = 0.728) the restoration. Males showed more hypersensitivity with Total-etch as compared to females before (p-value = 0.037), immediately after (p-value = 0.047), and 24 h after the restoration (p-value = 0.022). No significant difference was found between gender and Universal adhesive at all three stages (p-value > 0.05). The results suggest no significant difference in postoperative hypersensitivity between the two materials when good sample size and proper technique were observed along with the removal of bias like different patients having different pain perceptions and multiple operators having different operating skills. Males showed more hypersensitivity to Total-etch.

Trial registration number: Australian New Zealand Clinical Trials. Registry number: ACTRN12622001213730. (Retrospectively registered: 09/09/2022).

## Introduction

The revolution of esthetic dentistry was made possible by the introduction of adhesive restorations. Instead of opting for an aggressive tooth preparation, adhesive dentistry has made it possible for us to perform procedures in a more conservative and esthetic way^1^. The demand for esthetic restorations, the increased fear of amalgam toxicity, and the environmental problems associated with mercury have increasingly led to the use of resin composite for restorations^2^.

Postoperative Hypersensitivity (POH) after placing composite restoration has been a common problem experienced by patients worldwide. One of the most frustrating situations that dentists encounter is that after sending a happy patient back home with a beautiful restoration, they get a complaint from the same patient the very next day that the tooth is sensitive. No matter how stunning the restoration appears, the patient gets dissatisfied with the dentist because of the agonizing sensitivity he is experiencing^3^.

POH is caused by internal stress and polymerization shrinkage gaps that develop between the restoration and dentin which are more prone to leakage^4^. During mastication the accumulated fluid flows down the dentinal tubules, causing hypersensitivity^5^. The presence of bacteria and the communication between the dental pulp and oral cavity are further associated with this pulpal response^6^. This can be prevented by removing the bacteria-laden smear layer and sealing the dentinal tubules to prevent the ingress of new bacteria via microleakage. Total-etch adhesive resins are designed to provide a strong coupling between resin composites and enamel and dentin by the removal of the smear layer with 37% phosphoric acid. Mutual work of bonding agents, as well as a restorative material, can result in effective marginal sealing which can oppose contraction stress during the polymerization of composite^7,8^.

However, another school of thought is that in Total-etch, the exposed dentinal tubules by phosphoric acid are not completely sealed by bonding agents due to polymerization shrinkage or incomplete infiltration due to the presence of moisture. These exposed dentinal tubules are the source of hypersensitivity. This can be reduced by leaving the smear layer and not exposing the dentinal tubules^9^. A one-step Universal bond that contains Methacryloyloxydecyl dihydrogen phosphate (MDP), is introduced as a solution to this problem which does not remove the smear layer. Instead, it only modifies the smear layer^10^.

Authors who are in favor of less POH by Total-etch suggest that the acidic monomer in the Universal bond technique is not rinsed off from the surface of the tooth completely. The residual monomer would result in an increased risk of further demineralization and subsequent POH. Whether the single-step Universal adhesive seals the dentinal tubules adequately is also questionable. These adhesives are hydrophilic monomers and act as permeable membrane that allows water and fluids to move between the underlying dentin and interface^11^.

Total- etch is cheaper but is a multi-step technique as compared to the more expensive but single-step Universal bond technique. In other words, Total-etch is cheaper but Universal bond is a time-saving technique. Some dentists prefer to buy a low-cost bond and others want to save time. It becomes difficult for dentists to select between the two bonds because of the different beliefs about the bonds which still need clinical verification.

So, the rationale of the study was to remove the confusion among dentists regarding any difference in postoperative hypersensitivity between two bonds. The objective of the study is to find out if there is any statistically significant difference in POH after the application of a Two-step Total-etch and One-step Universal adhesive under composite restoration.

The null hypothesis of the study was “There is no significant difference in POH between Total-etch and Universal adhesive system before, immediately after, and 24 hours after the restoration”.

## Methodology

### Study design and setting

After obtaining approval from the ethical committee of the Rawal Institute of Health Sciences (RIHS), this randomized and parallel-designed clinical trial was carried out on progressively assigned 100 patients who visited the Department of Operative Dentistry, RIHS from 10/01/2019 to 21/08/2019. The CONSORT reporting guidelines were used for reporting parallel group randomized trials^12^.

### Sample size calculation

The sample size was calculated from the population means of two independent samples^3,13^ by using the WHO calculator. The values are given below:

Level of significance = 5%

Power of test = 80%

Population mean = 1.08^3^

The test value of population means = 0.79^13^

Pooled standard deviation = 0.38

Effect size = 1.08−0.79/0.38 = 0.763

Sample size = 100

Thus, the sample size calculated was 100 for each technique.

### Inclusion criteria

The patients who were willing to participate in the study were selected based on consecutive sampling. Patients of both genders between ages 16–60 years with at least two teeth with caries extended to the dentin i.e., ICDAS-4 (International caries detection and assessment system) but not more than one-half the distance between pulp and Dentino-enamel junction (DEJ) and requiring composite restoration were included in the study.

### Exclusion criteria

Patients having systemic diseases like uncontrolled diabetes, mentally handicapped, or with parafunctional habits such as bruxism, clenching teeth, etc. were excluded. Teeth with increased pre-operative sensitivity or non-vital teeth with pulp necrosis or irreversible pulpitis were excluded from the study. Deep carious lesions extended near the pulp were also excluded from the study to differentiate the symptoms of POH from the pulpal inflammation.

### Study instruments and method

A cold stimulus test was used to ascertain tooth vitality. Periapical radiographs were used to ensure that the carious lesion should not extend more than one-half the distance from the DEJ to the pulp i.e., ICDAS-4.

The teeth to be restored and the bonding to be applied were randomly selected by the patient himself by picking folded papers. The names of the bonds and teeth numbers were written on four different papers. Two papers with one bond name each were placed on one side and the other two papers with one tooth number written on each paper were placed on the other side. The patients were then requested to pick one folded paper from both sides. This defined the bond to be applied to the chosen tooth to be restored. The patients receiving treatment were blinded. Allocation concealment and sequence generation were applied as the patients randomly selected the bonds and teeth by picking the folded papers.

Informed consent was obtained from the patients who met the inclusion criteria and were willing to participate in the study, after briefing the aim of the study and its method. The restorations and VAS were performed by a single qualified operator i.e., a postgraduate trainee in Operative and Endodontics, to avoid bias due to technique sensitivity. Different patients have different pain thresholds e.g., moderate pain perceived by a person may be severe and unbearable for the other person. This comparative study was done on two teeth of the same patient by applying Universal adhesive (3M ESPE Scotch bond) on one tooth and Total-etch (3M ESPE Adper Single bond plus Adhesive) on another tooth. This minimizes the variation in pain perception.

Demographic details were obtained. Detailed history followed by clinical examination and pulp vitality test was taken. Patients were educated on marking the intensity of sensitivity of “test teeth” on the continuous visual analog scale (VAS, 0–10). The hypersensitivity noted before the start of the procedure was taken as a baseline and was noted on the patient proforma. The technique involved the application of cold spray i.e., Tetrafluoroethane (TFE) on a small piece of cotton. This TFE-soaked cotton was then applied to the mid-facial surface of the tooth to measure hypersensitivity. Extreme care was observed when preparing the cavity. Soft caries was removed manually by excavators. The removal of firm dentin and cavity preparation was done by using burs in the high-speed handpiece under coolant water flow followed by a cavity toilet. After cavity preparation, Total-etch adhesive was applied in the following way: 37% Phosphoric acid was applied for 25 s on the prepared cavity. The acid was then washed by air and water pressure through a triple syringe for 20 s. The surface was lightly air-dried. Then two coats of primer-bond solution were applied by scrubbing with a micro brush, for 20 s per coat. When the surface became shiny, it was light-cured for 20 s.

The other tooth of the same patient was restored by the application of Universal adhesive on the same day, in one sitting. The technique involved was preparing the cavity and applying two coats of Universal adhesive by scrubbing the entire preparation with a micro brush, for 10 s per coat. The surface was lightly air-dried to remove the residual solvent and light-cured for 20 s.

After curing the adhesives, the light-cured nano-composite (3M™ ESPE™ Filtek™ Z350 XT) was used for the restoration which was applied in increments (1–2 mm thick) by layering technique to reduce polymerization shrinkage in both techniques (Total-etch and Universal adhesive system). Each increment was light-cured for 20 s. POS was recorded immediately after the placement of the composite restoration. Follow-up was scheduled for the next day i.e., after 24 h to get the third reading of sensitivity. Later, the finishing was performed with carbide finishing bur, a polishing brush, and rubber cups by using polishing paste. The POS was recorded on a 0–10 VAS on all three occasions. In any event, the investigator refrained from administering local anesthesia because doing so might have influenced the sensitivity measurements taken immediately after the restoration. To maintain the pulp’s protection, teeth were chosen in which sound dentin was present between the pulp and cavity depth (ICDAS-4). Additionally, there was no requirement for local anesthesia when the affected dentin was removed using an excavator. An already-available, well-maintained Quartz tungsten curing light Sprectrum 800 (SP) (Dentsply DeTrey GmbH, Konstanz, Germany) was used in the curing of both adhesive systems and composite restorations.

There was no Patient or public involvement in designing or conducting the study. Determination of POH between two bonds was the primary outcome while determining the mean age and gender analysis for each bond was the secondary data output.

### Statistical analysis

The data was entered and analyzed in SPSS version 20.0. Descriptive analysis was used to determine the mean age. The normality of the data was checked by Kolmogorov-Smirnov Test. The data was found not normally distributed (p-value < 0.01). Therefore, the Mann–Whitney Test was applied to compare the VAS readings to determine the difference in POH between the two bonds as well as between gender and each bond. p-value < 0.05 was considered significant.

### Ethics approval and consent

All human studies have been reviewed by the ethics committee of the Rawal Institute of Health Sciences (RIHS/IRB 19-002) and have therefore been performed following the ethical standards laid down in an appropriate version of the Declaration of Helsinki. Verbal as well as written informed consent was obtained from each participant before the start of the procedure.

## Results

A total of 110 patients were assessed for eligibility. Seven patients did not meet the eligibility criteria and three did not come back after 24 h for reporting the 3rd reading. So, those patients were excluded from the study, as shown in the participant flow (Supplementary file). Out of the 100 patients enrolled, there were 33 (66 teeth) males and 67 (134 teeth) females. As shown in Table 1, no significant difference was found between postoperative hypersensitivity of the two adhesives before (p-value = 0.57), immediately after (p-value = 0.604), and 24 h after (p-value = 0.728) the restoration. According to Table 2, males showed more hypersensitivity with Total-etch as compared to females before (p value = 0.037), immediately after (p-value = 0.047), and 24 h after the restoration (p-value = 0.022). No significant difference was found between gender and Universal adhesive before (p-value = 0.588), immediately after (p-value = 0.305), and 24 h after (p-value = 0.830) the restoration, as mentioned in Table 3.

**Table 1:** Mann–Whitney Test to compare the VAS readings and to determine the difference in postoperative hypersensitivity between Total-etch and Universal adhesives.

	Groups	n	Median (Q2)	Interquartile range (IQR = Q3–Q1)	p-value
Baseline	Total-etch	100	2.0	5.0–0.0	0.573
	Universal	100	2.0	4.75–1.0	
Immediately after restoration	Total-etch	100	2.0	5.0–0.0	0.604
	Universal	100	2.0	4.0–0.00	
24 h after restoration	Total-etch	100	3.0	5.0–1.0	0.728
	Universal	100	3.0	5.0–1.0

**Table 2:** Mann-Whitney Test to compare the post-operative hypersensitivity of Total-etch adhesive with gender.

	Gender Males (n = 33) Females (n = 67)	Median (Q1)	Interquartile range (IQR = Q3–Q1)	p-value
Baseline	Males	3.5	6.0–2.0	0.037
	Females	1.5	5.0–0.0	
Immediately after restoration	Males	4.0	5.75–1.25	0.047
	Females	1.0	4.25–0.0	
24 h after restoration	Males	4.5	7.0–2.5	0.022
	Females	2.0	5.0–0.0

**Table 3:** Mann–Whitney test to compare the postoperative hypersensitivity of Universal adhesive with gender.

	Gender Males (n = 33) Females (n = 67)	Median (Q1)	Interquartile range (IQR = Q3–Q1)	p-value
Baseline	Males	2.00	3.75–1.00	0.588
	Females	2.00	0.00–1.00	
Immediately after restoration	Males	2.00	3.00–0.00	0.305
	Females	2.00	0.00–0.00	
24 h after restoration	Males	3.00	4.00–2.50	0.830
	Females	2.50	0.00–0.00	

Descriptive analysis showed that the mean age of patients was 30.96 ± 10.3. with a minimum age of 16 and a maximum age of 58, as shown in Table 4. There was no harm to anyone during this study.

**Table 4:** Descriptive analysis to determine the mean age.

	N	Minimum	Maximum	Mean	Std. deviation
Age	100	16	58	30.96	10.3

## Discussion

Postoperative hypersensitivity is frequently associated with composite restorations. After in-vitro testing of the new dentin-bonding agent i.e., Universal adhesive, it was necessary to evaluate its clinical efficacy. Unfortunately, it is still without sufficient clinical evidence^14^.

The current study suggests no significant difference between the two systems at any time i.e., before, immediately after, and 24 h after the restoration. According to a thorough literature search, no such comparison of POH has been published up to this point between the gold standard Total-etch adhesive and the latest Universal adhesive system. So, no benchmark study is available for comparison. However, there are certain published reviews and meta-analyses describing the characteristics of these adhesives. Moreover, there are some studies to find the difference in POH concerning the different modes of application of these adhesives and, by different operators with different operating skills. These studies are referred to support the findings of the current study.

The results of the present study contrast with a study by Toshniwal N about the characteristics of different generations of the adhesive system. The mentioned study suggested that the Total-etch bond causes more hypersensitivity as compared to the Universal bond technique^1^. Other reviews and meta-analyses also suggested that the Universal adhesive system causes less hypersensitivity^15–17^ which conflicts with the present study.

The mode of application of the Universal bond in the current study was ‘‘Self-etch.’’ Multiple studies comparing the POH of different modes of application of Universal adhesive suggested no significant difference. This means that any mode of application of Universal bond can be used under composite restoration without fear of POH^18,19^. Another investigation demonstrated that the POH of the Universal adhesive in the Self etch and Selective enamel etch modes was much lower than in the Total-etch application method^20^.

An in vivo study had the limitation of subjective variations in reporting postoperative sensitivity^17^. The bias is controlled in the current study by comparing the hypersensitivity between two bonds by applying each to the different teeth of the same patient.

According to a study by Sancakli HS., the POH scores for Total-etch were higher for the dental students (p-value < 0.05), while the POH scores for Universal bond did not differ significantly for the operators (p-value > 0.05)^12^. The POH was significant in the mentioned study because different operators were doing the restorations, i.e., due to the difference in operating skills. This bias is controlled in the current study by restoring all teeth by a single operator.

Gender analysis of the current study suggested no difference in POH with Universal adhesives. The results are the same as a study by Francis et al., which suggested no significant difference in POH of Universal adhesive application between males and females^17^. However, the present study suggested that males were more hypersensitive to Total-etch as compared to females.

The present study addressed the POH between the two bonds before, after, and 24 h after the restoration. However, it did not report a long-term follow-up due to resource constraints and lack of patients’ commitment toward follow-up which is a way forward for future researchers. Nevertheless, proper technique, single operator, good sample size, and application of both adhesives on two teeth of the same patient in the current study, remove the confounding factors that help in determining the true comparison of POH between the two bonds. The present study retained the null hypothesis that there is no difference in POH between the Total-etch and Universal adhesive system under composite restoration at any time, after controlling the confounding factors.

## Conclusion and practical implication

The current study found no significant difference in hypersensitivity between the gold standard Total-etch and the latest Universal adhesive system when the proper technique of placement of restoration was keenly observed. Thus, dentists can choose any of the two bonds under composite restoration according to their convenience.

### Supplementary Information

Supplementary Information 1.

Supplementary Information 2.

## Data Availability

The datasets used and analyzed during the current study are available from the corresponding author upon reasonable request.
