# Supplementary material for: Comparison of postoperative hypersensitivity between Total-etch and Universal adhesive system: a randomized clinical trial
Source: Sci Rep. 2024 Jan 5;14:678. doi: 10.1038/s41598-024-51175-8 (PMC10770022; doi:10.1038/s41598-024-51175-8)
Supplement: Supplementary file 1 — Supplementary Information 1. [file 41598_2024_51175_MOESM1_ESM.docx]

**PARTICIPANT FLOW**

Assessed for eligibility (n= 110)

Excluded (n= 10)

♦  Not meeting inclusion criteria (n= 7)

♦  Did not come back after 24 hours (n= 3)

Analysed (n=100)

Male (n=33)

 Female (n=67)

Patients who came back for follow up (n= 100)

Patients who did not come back for follow up (n= 3)

Teeth restored with total etch technique

(n= 103)

Patients who came back for follow up (n= 100)

Patients who did not come back for follow up (n= 3)

Teeth restored with Universal bond technique (n= 103)

Analysed (n= 100)

Male (n=33)

 Female (n=67)

## Allocation

## Analysis

## Follow-Up

Allocated (n= 103)

## Enrollment

## 2. Baseline characteristics
