# Supplementary material for: Comparison of postoperative hypersensitivity between Total-etch and Universal adhesive system: a randomized clinical trial
Source: Sci Rep. 2024 Jan 5;14:678. doi: 10.1038/s41598-024-51175-8 (PMC10770022; doi:10.1038/s41598-024-51175-8)
Supplement: Supplementary file 2 — Supplementary Information 2. [file 41598_2024_51175_MOESM2_ESM.docx]

**Research Proforma Appendix C**

Comparison of post-operative hypersensitivity between total-etch and Universal bonding technique, A randomized clinical trial

Serial #________________________________________________________

Name: ________________________________________________________

Age/ Sex: ______________________________________________________

MR #: ________________________________________________________

Address: _______________________________________________________

Contact #: ______________________________________________________

Date: __________________________________________________________

Total-Etch Tooth #: ______________________________________________

Universal bond Tooth #: _______________________________________________

Next Appointment Date: ___________________________________________

Note:

Visual Analog Scale (VAS)


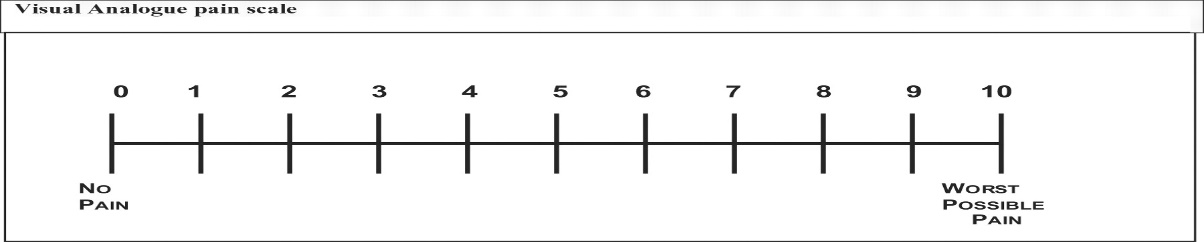


VAS for Total-etch

Before restoration ______

After restoration ______

24 hours after restoration ­­______

VAS for Universal bond

Before restoration ______

After restoration ______

24 hours after restoration ­­ ______
